# Commentary: Insulin-Producing Organoids Engineered From Islet and Amniotic Epithelial Cells to Treat Diabetes

**DOI:** 10.3389/fendo.2020.546114

**Published:** 2020-10-06

**Authors:** Lorenzo Cobianchi, Beat Moeckli, Stefania Croce

**Affiliations:** ^1^Department of General Surgery, Fondazione IRCCS Policlinico San Matteo, Pavia, Italy; ^2^Department of Clinical, Surgical, Diagnostic & Pediatric Sciences, University of Pavia, Pavia, Italy; ^3^Department of Surgery, University of Geneva, Geneva, Switzerland

**Keywords:** stem cells, surgery, diabetes, organoids, organ replacement

Islet transplantation has been an option for treating diabetes since the 1970s (1). Over the years, this procedure has been established as one of the most relevant examples of cell transplantation for the purpose of function replacement. However, during the same time we have also documented the limits of this procedure (2, 3). It is too early to say that islet transplantation is on the verge of becoming standard-of-care. We are still facing many hurdles before establishing beta cell replacement as a reliable long-term therapy. Fortunately advances in the field of regenerative medicine, stem cell research, and tissue engineering made it possible to significantly improve beta islet cell replacement therapy (4, 5). The urge to develop new models of regenerative medicine has led to the creation of new platforms like organoid engineering. This novel strategy is based on the use of multicellular, organized, three-dimensional (3D) structures that present specific organ functions. Organoids can be obtained from different stem cells that spontaneously organize into structures containing functional cell types or progenitors. In this way, organoids are able to mimic an organ’s *in vivo* structure and complexity. For these reasons, we believe that organoid techniques will be useful for different clinical applications, such as disease modeling, drug screening, and also in a therapeutic aim.

The 3D cell aggregates system (3DCAgg) is a highly promising technology that has already been used to investigate diseases of the central nervous system (6). Lebreton et al. describe the use of human amniotic epithelial cells (hAECs) in the construction of functional insulin-secreting organoids in their article “Insulin-producing organoids engineered from islet and amniotic epithelial cells to treat diabetes” (7). There are two relevant aspects to this publication: the application of 3DCAgg and the use of hAECS.

Recently, several studies have demonstrated that mature pancreatic cells or stem cells can generate functional pancreatic organoids (POs) (8). However, these organoid systems have limitations, such as the difficulty of producing an organoid with a specific shape and size, the inability to recreate *in vitro*, the lack of a vascular system (9, 10), the presence of undifferentiated cells in organoids, and the difficulty in obtaining long-term glycemic control. Previous attempts in maintaining normoglycemia for more than 12 days after implantation of organoids, comprised of only islet cells (IC), in diabetic mice failed (11). In this regard, Lebreton et al. made a breakthrough in organoid research by generating functional POs from two types of epithelial derived cells: dissociated islet cells and hAECs. This study is valuable for several reasons. Firstly, by mixing a specific number of ICs and hAECs, Lebreton et al. grew 3D organoids into predictable shapes and sizes, obtaining morphology and dimensions that are equivalent to those of a pancreatic islet. Secondly, the authors have highlighted the importance of adding an accessory cell component to the dissociated islets. In fact, hAECs increased the protection of the 3D structure under the inflammatory and hypoxic conditions of the pre-implantation period. The organoid shape, cell–cell adhesions mediated by E-CAM, and paracrine signaling generated by hAECs seem to be fundamental for promoting the differentiation of insulin-producing cells into POs, as opposed to ICs-spheroids without an additional component. Moreover, the functional POs generated *in vitro* were able to secrete glucagon, somatostatin, and insulin in response to high glucose levels and restored normoglycemia after the implantation in diabetic mice.

Another very important aspect of this work is the effect of hAECs in inducing new blood vessel formation. In fact, hAECs appears to promote endothelial cell proliferation and angiogenesis in SCID mice by secreting soluble factors. In accordance with these findings, it has been shown that hAECs can be used to improve the long-term viability and function of islets (cytoprotective effect) in both *in vitro* and *in vivo* studies. For these reasons, hAECs seem to be a promising option for organoid engineering in the field of diabetes research.

Currently, the possibility of using organoids in cell therapy is encouraging, but further validation is required before organoids can be used in the clinical setting. The process of generating organoids still harbors a random component. The reproducibility of the experimental setting is limited, making the step from bench to bedside challenging. Moreover, similar to whole organ pancreas or islet transplantation, POs activate the host immune system, leading to loss of function and cell death in immunocompetent mice. Therefore, the main issue that remains to be solved is the post-transplantation islet loss due to the host immune system. Overcoming these challenges will require a multidisciplinary approach. Currently, the field of bioengineering has developed promising new micro- and macro-encapsulation strategies (12, 13). For example, nanotechnology research is focused on the generation of POs by using different polymers and ECM scaffolds as encapsulating platforms in order to control the localization of POs and protect them from the host immune system (14). Furthermore, bioreactors could be helpful in generating and/or maintaining viable and functional POs and produce large amounts of organoids. Additional studies are needed to standardize the methods and control for these aspects.

In summary, organoid technology represents a powerful tool that is suitable for personalized medicine. In particular, the generation of insulin-secreting organoids is a very promising strategy in the field of regenerative medicine. In the present work, Lebreton et al. have developed a successful protocol to increase the efficacy and functionality of POs. Their results offer hope that POs will one day provide an alternative treatment for diabetes. In the coming years, further efforts are needed to achieve a successful clinical application. We are convinced that organoid technology deserves to be widely explored in order to obtain treatment alternatives for several diseases including diabetes.

## Author Contributions

LC had the idea and wrote the manuscript together with SC and BM. All authors contributed to the article and approved the submitted version.

## Conflict of Interest

The authors declare that the research was conducted in the absence of any commercial or financial relationships that could be construed as a potential conflict of interest.
